# Fructose fuels lung adenocarcinoma through GLUT5

**DOI:** 10.1038/s41419-018-0630-x

**Published:** 2018-05-10

**Authors:** Yuanyuan Weng, Jin Zhu, Zhenhong Chen, Jingqi Fu, Feng Zhang

**Affiliations:** 1 Core Facility, Department of Clinical Laboratory, Quzhou People’s Hospital, Quzhou, Zhejiang China; 2 Department of Oncology, Quzhou People’s Hospital, Quzhou, Zhejiang China; 3 Program of Environmental Toxicology, School of Public Health, China Medical University, Shenyang, China

**Fig. 1:**
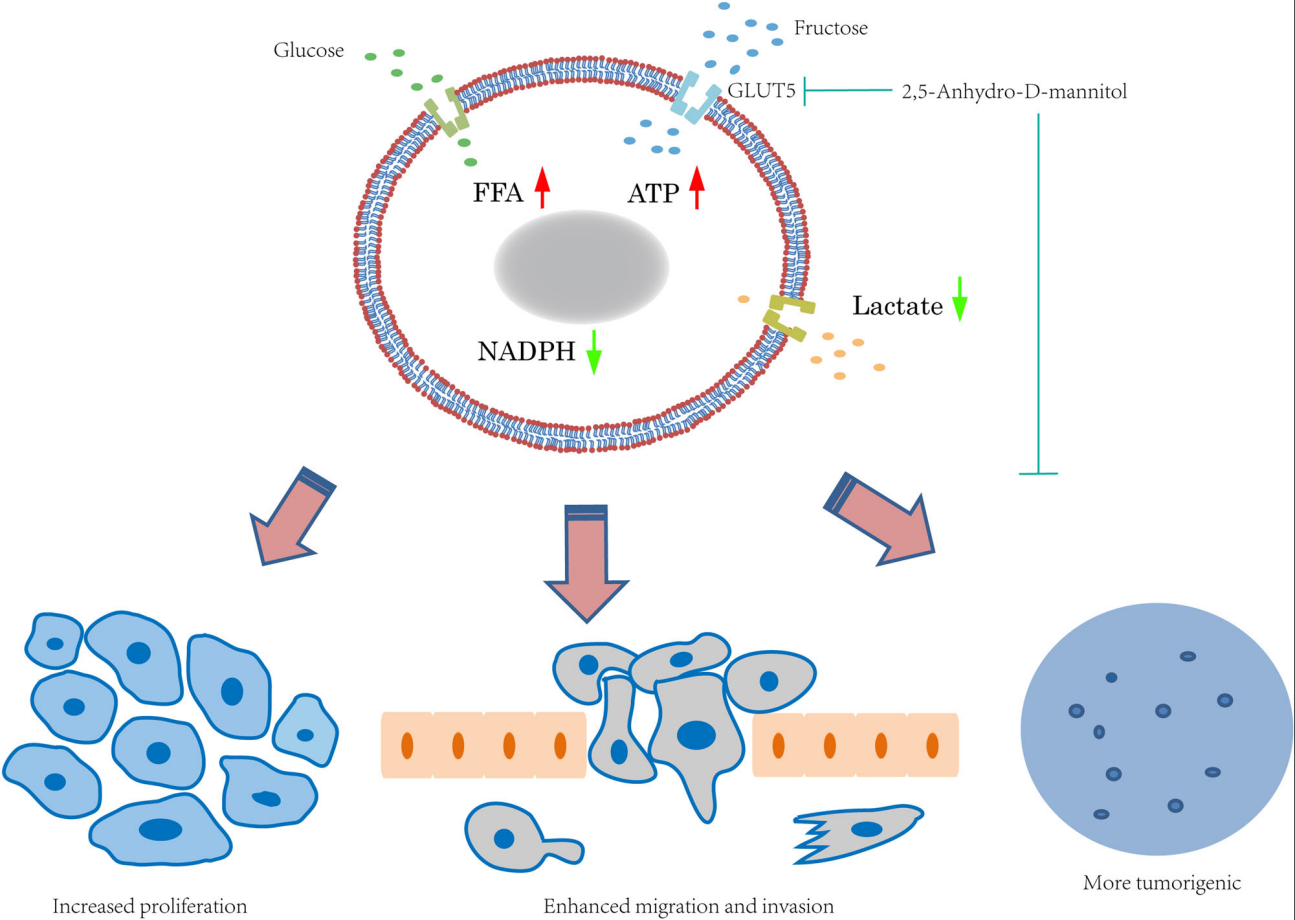
Fructose promotes lung adenocarcinoma cell survival and metastasis through GLUT5. LUAD cells utilize both glucose and fructose as nutrients for energetic and biosynthetic purposes. Especially, when glucose is scarce, fructose may function as a crucial alternative. Fructose, ingested through GLUT5, could efficiently enhance cancer cell proliferation, migration, invasion, and tumorigenicity. Compared to glucose, fructose metabolism facilitates intracellular FFA accumulation and ATP production while decreases NADPH and lactate in LUAD cells. The green arrow indicates that fructose produces less metabolites than glucose under the same concentration, while the red arrow indicates the opposite trend. *FFA* free fatty acid, *2,5-Anhydro-D-mannitol* fructose analog with high affinity for GLUT5

Fructose is one of the most common dietary carbohydrates, accounting for ~5–15% of daily calorie intake. Because of its lower glycemic index compared to glucose, fructose has been utilized for a long time as a food sweetener for serving people with diabetics, children, and elderly^1^. In the last decade, strong attention has been drawn to focus on the influence of fructose consumption on many diseases, including tumorigenesis, because of its large intake world widely. Biologically, following the intestinal ingestion, most of the food-origin fructose is converted into glycogen and fat in liver tissue, while the rest enters bloodstream for the direct metabolism in other organs. Unlike glucose, of which concentration is tightly controlled through endocrine signaling, the level of serum fructose fluctuates from micromole to millimole per liter, largely depending on the amount of intake^2^. Data on epidemiological survey have demonstrated that conditions including obesity, diabetes, as well as heart and kidney diseases are correlated with long-term high-fructose intake, which is commonly seen in various modern diets^2^. (Fig. 1)

In the field of cancer research, high level of food-intake fructose has been thought to be related to metabolism-induced cancer; yet, the details of direct link between fructose metabolism and cancer have only been able to slowly unbury along with the recent progression on the molecular/cellular basis on cancer metabolism. A study by Jiang et al. has shown that fructose increases the risk of breast cancer progression and metastasis by inducing the productions of lipoxygenase-12 and a related fatty acid 12-HETE in breast cancer cells^3^. In pancreatic tissue, it has been confirmed that fructose can be utilized for the synthesis of nucleic acid and to promote cell proliferation by cancer cells^4^. Fructose has also been identified as an alternative source for energy and biomaterial production by acute myeloid leukemia (AML) cells, under the condition of low blood glucose^5^.

In particular within our scope of interest, lung cancer, our recent published study in *Cell Death and Discovery* has uncovered the crucial role of fructose intake on enhancing the growth and metastasis of lung adenocarcinoma (LUAD) cells^6^. In this study, fructose and glucose, under the same concentration, exhibit distinct efficiencies on promoting LUAD cell proliferation in vitro. Within the examined cell lines, the enhancing effects of fructose on cell proliferation were measured weaker in comparison to that caused by glucose, in each of the cell model. The required concentration for fructose to induce the peak effects on cell phenotype is significantly higher than that of glucose, indicating the differences in cellular uptaking and/or utilization of fructose or glucose. Moreover, fructose and glucose are differentially metabolized under our in vitro experimental conditions. When supplied with fructose, LUAD cells synthesized higher levels of free fatty acids and ATP, compared to glucose-treated groups. Likewise, distinctives in metabolizing these two carbohydrates have been reported in previous finding that, in liver tissue, the catabolism of fructose that bypasses important glycolytic regulatory steps in glycolysis leads to greater lipogenesis than that from glycolysis-regulated lipid production^7^. Our results on the identification of differential metabolic patterns on the usage of fructose and glucose by LUAD cells provides valuable insights on the cellular favor of energy and biomass production to sustain themselves upon the substrate availability, i.e., serum level(s) of fructose and/or glucose. Such evidence furthers the elucidation on the survival strategy of tumor cells under different circumstances with varied ingestions of nutrients.

In terms of molecular basis of fructose metabolism, enzymes including fructose-transporting protein family (SLC2a or GLUT), ketohexokinase (KHK), and aldolase have been identified to be involved in this process. GLUT2, 5, 7, 8, 9, and 11, members of the SLC2A family, locating on the plasma membrane, facilitate the transportation of fructose. GLUT2, 7, 8, 9, and 11 also transport other small molecules in addition to fructose, whereas GLUT5 is strictly for fructose only^8^. In normal physiological conditions, fructose metabolism is enzymatically related to the availability of fructose transporter and ketohexokinase activity. However, in pathological conditions, i.e., tumor, the knowledge on the mechanism responsible for fructose metabolism remains very limited. GLUT5, encoded by the *SLC2A5* gene, has much higher affinity to fructose rather than other carbohydrates including glucose and galactose. Normally, GLUT5 is not detectable in normal mammary cells, yet, in breast cancer tissue, elevated *SLC2A5* mRNA levels together with higher fructose uptake were reported by Zamora-Leon et al.^9^. In another study, within tumor cells from patients with AML, increased GLUT5 expressions were confirmed and the severity of pathological progression was demonstrated associated with the level of GLUT5^5^. In both cases, the cellular uptake of fructose and tumor cell proliferation were all significantly inhibited by the elimination of GLUT5 in breast cancer cells or AML cells, respectively^5, 9^.

Our recent data in *Cell Death and Discovery* show that the expression of *SLC2A5* is upregulated in non-small cell lung cancer (NSCLC) samples compared to normal lung tissue. The overexpression of *SLC2A5* is closely related to the poor prognosis of LUAD. Experiments with isotope-labeled fructose incorporation reveal the linear correlation between the uptake efficiency of fructose and the mRNA level of *SLC2A5*. Increasing or inhibiting the expression of *SLC2A5* in the cells resulted in a corresponding change in fructose uptake. As we known, the metabolism of cancer cells is largely dependent on the tumor-associated microenvironment. Despite our evidence that the interruption on the transcription of *SLC2A5* by short hairpin RNA directly inhibits the proliferation and at the same time induces the apoptosis of LUAD cells in vitro, the exact role of GLUT5 on the progression of NSCLC in vivo is remained to be confirmed. To address this issue, studies on observing the initiation and development of NSCLC employing GLUT5 conditional knockout mice fed with high-fructose diet or wild-type mice inoculated with LUAD cells lacking GLUT5 are rationale to uncover the function of GLUT5 in a physiological context. In addition, the metabolic features of NSCLC are highly heterogeneous within and between tumors. The contribution of non-glucose nutrients to tumor progression varies between well-perfused and poor-perfused tumor regions^10^. Experiments of using ^13^C isotope-labeled fructose perfusion to detect the actual utilization of fructose in animal models or patients are predicted to provide important clinical data.

In regard to the regulation on GLUT5, the major fructose transporter, numerous lines of work have been conducted to contribute to the pathway-mapping. Glucocorticoid hormone dexamethasone has been previously shown to promote *SLC2A5* expression by inducing the translocation of nuclear glucocorticoid receptor and the epigenetic modifications of histone proteins^11, 12^. Long-term insulin treatments on L6 muscle cells induce GLUT5 protein productions in a dosage-dependent manner^13^. Intestinal *SLC2A5* mRNA level is rapidly increased with weaning independent of diet and its expression is further induced by diets containing fructose^2^. Fructose stabilizes GLUT5 mRNA in differentiated Caco-2 cells through the cAMP pathway and the binding on PABP-interacting protein 2 (Paip2)^14^. Introduction of fructose into the lumen of adult wild-type mice elevates mRNA and protein levels of GLUT5^15^. Carbohydrate-responsive element-binding protein, a transcription factor that is sensitive to intracellular carbohydrate nutrients, regulates intestinal GLUT5 expression and it is essential for systemic fructose tolerance^2^. Nonetheless, the details on the regulation of *SLC2A5* in lung cancer are yet to be uncovered. Our results reveal that neither the known cancer driver genes nor the extracellular fructose level is efficient to influent *SLC2A5* expression in LUAD. Within the tissue samples from cancer patients we screened, the transcriptional levels of *GLUT5* are trending to be associated to the events of activating tumor-driven genes, i.e., *EGFR* mutation or *ALK* fusion. However, similar molecular interaction is yet to be reproduced in vitro in order to fully address the comprehensive cellular signaling pathway. Such limitations on the current study indicate the demands on further investigations that more patient samples are required to confirm the relationship between cancer driver genes and GLUT5 abundance; moreover, besides of cancer driver genes, extracellular-microenvironment conditions including hypoxia, hypoglycemia, and cytokines are not to be omitted when interpreting the in vivo regulatory effects on GLUT5. Furthermore, in addition to the regulation of GLUT5 abundance, the intracellular and membrane distribution of GLUT5 possibly tuning by the existing similar mechanisms on GLUT1 add another complex layer to the full picture. To summarize, we suggest that further studies on GLUT5 biology in tumor cells including the transcriptional/translational control of the gene and the cellular distribution of the protein production are strongly warranted.
